# A new approach to measure the resistance of fabric to liquid and viral penetration

**DOI:** 10.1371/journal.pone.0211827

**Published:** 2019-02-08

**Authors:** Min Li, Jennifer L. Furlong, Patrick L. Yorio, Lee Portnoff

**Affiliations:** 1 Department of Chemistry and Physics, California University of Pennsylvania, California, PA, United States of America; 2 National Institute for Occupational Safety and Health, National Personal Protective Technology Laboratory Pittsburgh, PA, United States of America; Fondazione Istituto Italiano di Tecnologia, ITALY

## Abstract

Protective clothing manufacturers routinely test their products for resistance to liquid and viral penetration. Several of the test methods specified by the American Society for Testing and Materials (ASTM) and the International Organization for Standardization (ISO) for penetration testing produce binary results (i.e. pass or fail), deliver imprecise pressure regulation, and do not record the location at which penetration events occur. Instead, our approach measures a continuous variable (time of penetration) during a slow and continuous increase of hydrostatic pressure and retains the location of penetration events. Using a fluorescent dye to enhance visual detection, we evaluate temporal and spatial patterns of penetration events. We then compare the time of liquid penetration with the time of penetration of two bacteriophages (Phi-X174 and MS2). For the fabric tested, the mean viral penetration occurred 0.29 minutes earlier than liquid penetration when solved by logistic regression. The breakthrough time of MS2 was not different from the Phi-X174 bacteriophage. The time of liquid penetration was a latent indicator of the time of viral penetration.

## Introduction

Healthcare personnel (HCP) have an increased risk of exposure to disease from close contact with infected patients [1]. During medical procedures and routine visits, pathogens contained in a patients’ body fluids (e.g. blood, saliva, urine, feces, or vomit) may lead to infection of HCP. One possible means of exposure to infectious body fluid is by penetration through protective clothing (e.g., surgical gowns).

In 1945, the American Association of Textile Chemists and Colorists (AATCC) devised a test that measures the resistance of fabrics to the penetration of water by impact [2]. For this test, a stream of water drips onto a fabric specimen that covers a piece of blotter paper. The mass of water absorbed into the paper indicates the amount of water penetration. In 1968, AATCC devised a more challenging test that used hydrostatic pressure to evaluate fabrics that outperform the 1945 test [3]. The hydrostatic test applies increasing pressure to a fabric specimen in a penetration cell. The pressure required to cause penetration indicates the hydrostatic resistance of the fabric.

In 1991, the Occupational Safety and Health Administration (OSHA) published its first bloodborne pathogen standard [4]. The standard required employers to provide viral protection appropriate for “the task and degree of exposure anticipated.” However, the standard did not select a test method for this requirement. An AATCC committee had worked to create a universally accepted penetration test, but the standards board terminated before reaching agreement [5]. ASTM took a unilateral approach and proposed a dichotomous (i.e. pass/fail) test challenging fabric specimens against 2 psi of hydrostatic pressure. In 1992, ASTM published two emergency standards, ES21 and ES22 [6] [7]. These standards were based on the ASTM F903 test method [8]. ES21 evaluated the resistance to penetration by synthetic blood, while ES22 evaluated the resistance to penetration by Phi-X174 bacteriophage in a liquid nutrient broth. In 1995, ES21 and ES22 became permanent ASTM standards (F1670 and F1671) [9, 10].

To classify protective apparel and evaluate performance, the Association for the Advancement of Medical Instrumentation (AAMI) incorporated ASTM F1670 and ASTM F1671 with two additional tests (AATCC 42 and AATCC 127) into their PB70 standard [11]. Also, the International Organization for Standardization (ISO) adopted and modified ASTM F1670 and ASTM F1671 into ISO 16603 and 16604 by adding six levels of pressure for the purpose of ranking garments into classes (Table 1) [12, 13]. In general, PB70 tests not relate to EN tests and manufactures therefore need to duplicate garment performance testing for sales in US and European markets.

**Table 1:** American National Standards Institute (ANSI) / Association for the Advancement of Medical Instrumentation (AAMI) PB70 liquid barrier performance and classification (A) and European Standards (EN) performance requirements (B).

**A) ANSI/AAMI PB70**		
**Rank**	**Test**	**Criteria**
**Level 1**	AATCC 42	< = 4.5 g
**Level 2**	AATCC 42	< = 1.0 g
	AATCC 127	> = 20 cm (1.96 kPa)
**Level 3**	AATCC 42	< = 1.0 g
	AATCC 127	> = 50 cm (4.90 kPa)
**Level 4**	ASTM F1670	pass 2 psi (13.8 kPa)
	ASTM F1671	pass 2 psi (13.8 kPa)
**B) EN 14126:2003**		
**Rank**	**Test**	**Criteria**
**Class 1**	ISO 16603 & 16604	0 kPa
**Class 2**	ISO 16603 & 16604	1.75 kPa
**Class 3**	ISO 16603 & 16604	3.5 kPa
**Class 4**	ISO 16603 & 16604	7.0 kPa
**Class 5**	ISO 16603 & 16604	14.0 kPa
**Class 6**	ISO 16603 & 16604	20.0 kPa

ASTM F1670 and ASTM F1671 provide clear delineation between materials that are protective and those that are not, however, these tests provide a poor means to differentiate fabric performance [14]. ISO 16603 and ISO 16604 provide a rank of fabric performance; however, garment failure can span multiple classes or differ from one testing laboratory to another. Only AATCC 127 provides a continuous variable (pressure) that may show a complete profile of sample failures. However, AATCC 127 provides a haphazard spatial analysis that includes all penetration events at the perimeter, regardless of whether the apparatus caused the failures [3].

Another concern is that several tests (ASTM F1670, ASTM F1671, ISO 16603, and ISO 16604) do not accurately account for pressure delivery. The vertical orientation of the penetration cell adds gravitation head pressure (98 Pa per centimeter of liquid). Furthermore, the tolerance of the manual pressure regulator used in ASTM F1670 and ASTM F1671 delivers 2,758 Pa (±0.2 psi) of variability.

Our approach is to challenge fabric under a slow and continuous increase of hydrostatic pressure and to evaluate temporal and spatial patterns using a fluorescent dye to enhance visual detection. This approach is similar to the AATCC 127 test method [3]. However, our pressure regime begins with 5 minutes of no pressure followed by a pressure increase six times slower than the AATCC test. Longer exposure to the liquid can better simulate real-life conditions of extended wear. Another difference is that AATCC 127 specifies water, whereas our test allows for testing water, synthetic blood, nutrient broth, and other liquids that have differing degrees of penetrability. However, most importantly, our method includes a viral test that is absent from AATCC 127. Video recordings retain the time and location of penetration events, a mass-flow controller delivers accurate pressure regulation, and spatial analysis provides a means to evaluate additional pressure from gravitational head.

ASTM and ISO test methods (ASTM F1670, ASTM F1671, ISO 16603, and ISO 16604) specify a quick visual screening test and a laborious viral penetration test, however, the difference between liquid and viral penetration is not clear. Shimasaki et. al. demonstrated that the amount of virus was proportional to the volume of carrier liquid that penetrated [15]. By showing a correlation between liquid and viral penetration, Shimasaki suggests that liquid penetration may be a latent indicator of viral penetration. The Shimasaki technique, however, only accounts for the viruses after liquid penetration already occurs. The techniques presented in this paper complement Shimasaki’s technique by evaluating the time of viral penetration before it becomes visible.

The purposes of this study are 1) to evaluate a new approach to measure liquid and viral penetration; 2) to provide temporal and spatial analysis of liquid penetration; 3) to compare the breakthrough time of liquid and viral penetration; and 4) to compare the breakthrough time of two different viruses Phi-X174 and MS2. This paper is part of a larger study to improve the scientific understanding of protective clothing test methods [16, 17].

## Materials and methods

### Test apparatus

**Fig 1:**
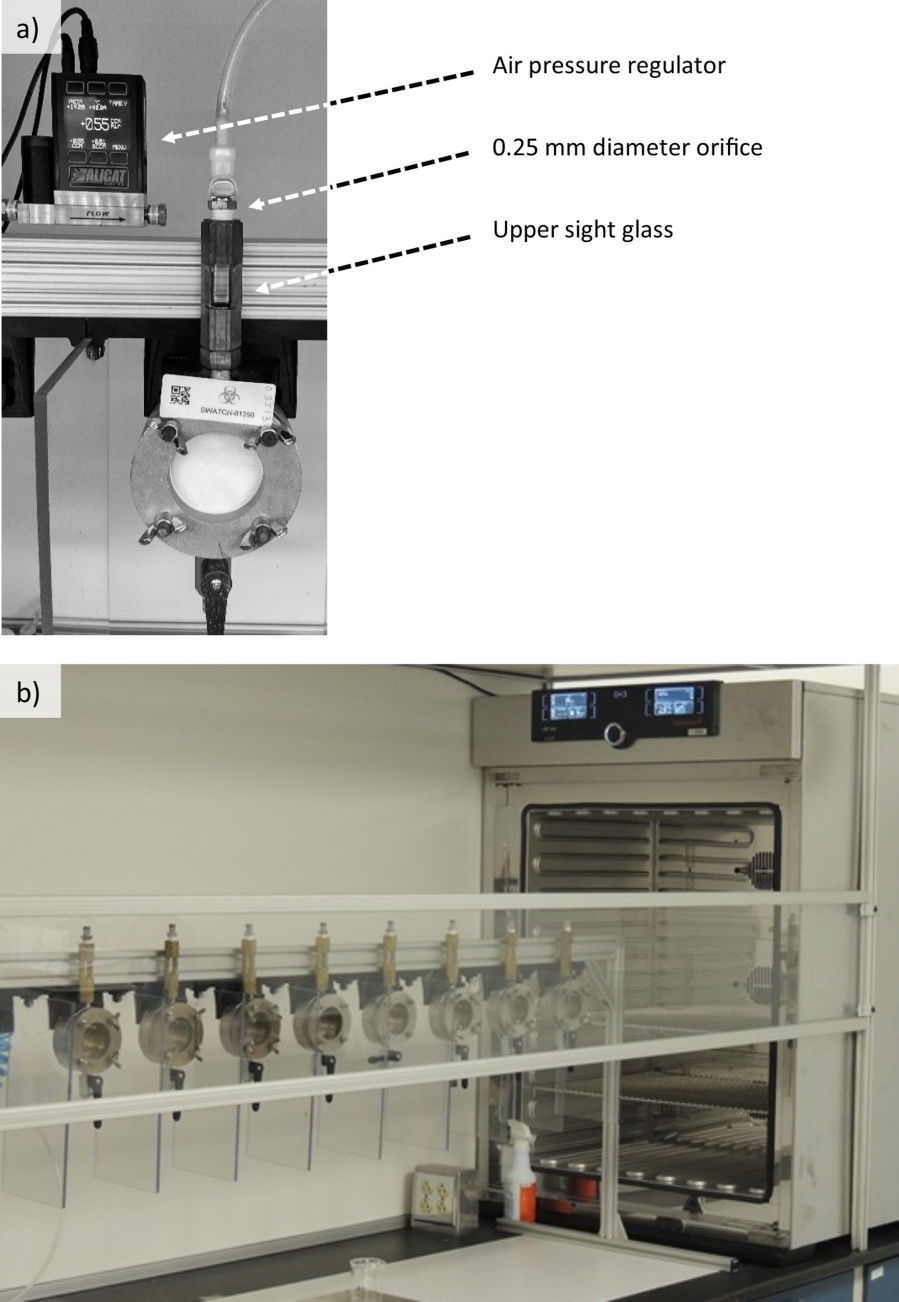
Liquid penetration test assembly: a) modified ASTM F903 penetration cell, and b) array of eight penetration cells inside the temperature and humidity-controlled chamber.

All liquid and viral penetration tests in this study were conducted using a penetration apparatus based on the ASTM F903 standard [8]. Custom modifications include the following: two mass-flow regulators to provide pressure control within 1%, a 0.25 mm diameter orifice to maintain pressurization if adjacent cells depressurized during a test, and an upper sight glass to provide visual indication of the fluid level. An array of eight penetration cells were enclosed within a climate controlled chamber (Fig 1A). Temperature and humidity were controlled by an incubator (Model: 260plus, Memmert, Germany) enclosed within a chamber (Fig 1B) that was closed to atmosphere by vinyl strip doors (Model: H-3202, Uline, Pleasant Prairie, WI).

The primary mass flow control regulator (Model: MC-5SLPM-D, Alicat Scientific, Tucson, AZ) was configured to pressure regulation mode. A secondary regulator (Model: MC-50SCCM-D, Alicat Scientific, Tucson, AZ) was added to provide 4 cc/min nominal airflow through the primary regulator. Pressure was validated with a NIST traceable manometer to be within 1% from 0 to 90 kPa.

### Fabric specimens

**Fig 2:**
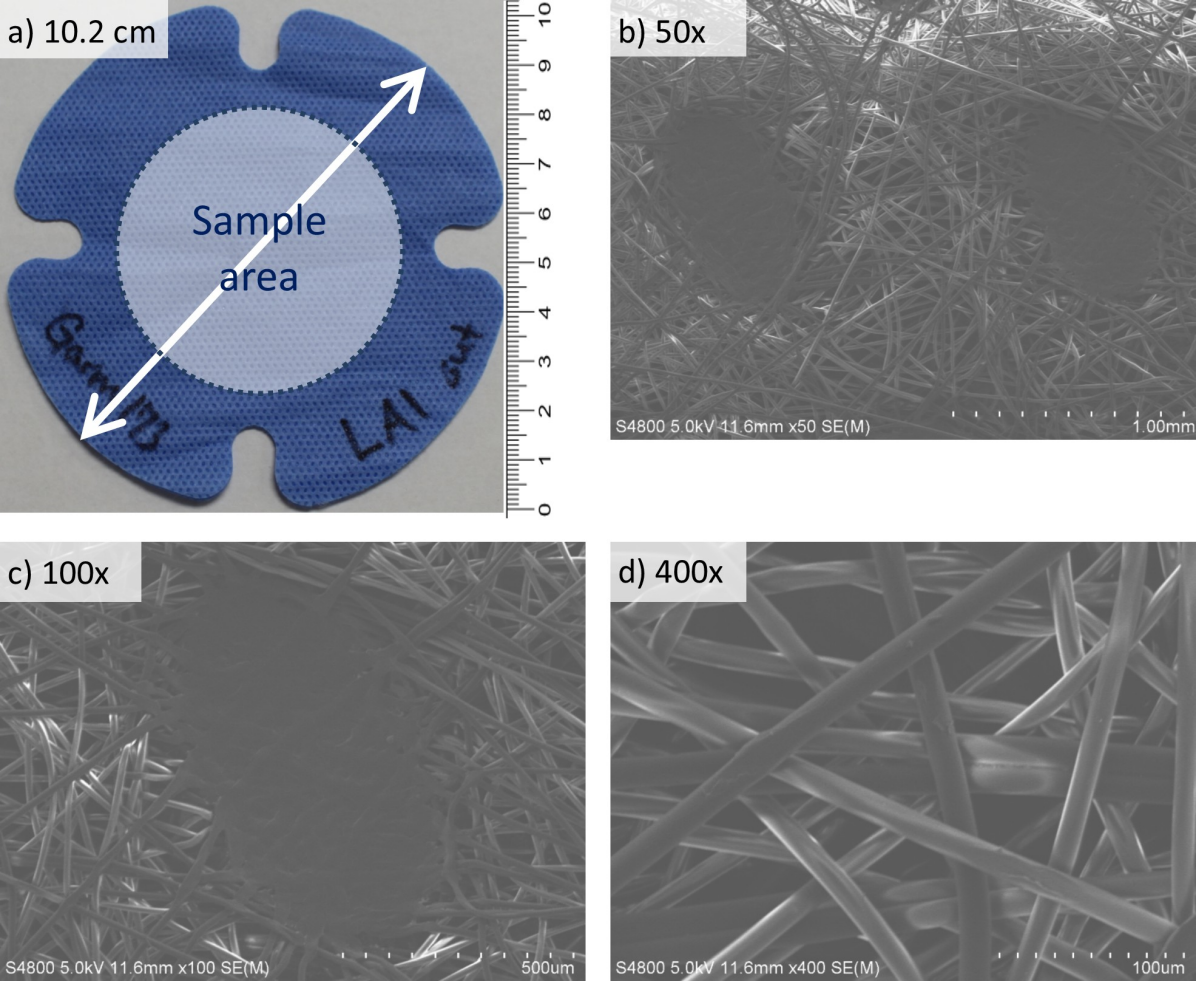
ANSI/AAMI Level 3, five layer, non-woven, spunbond and melt blown surgical gown specimen: a) exterior side having a 10.2 cm outer diameter and a 25.65 cm^2^ sample area, b) 50x magnification, c) 100x magnification, and d) 400x magnification.

This paper is part of a multi-dimensional comparison of ten garment models, four test liquids, and factors of pre-wetting, apparatus screen, and elevated temperature and humidity. For clarity, this manuscript details methods and highlights only one garment model: an ANSI/AAMI Level 3, five layer, non-woven, spunbond and melt blown surgical gown (Fig 2). Top layer fibers, measuring 10–15 microns diameter appear to be heated and compressed into oval indentations (Figs 2B, 2C and 2D).

Twenty-six gowns were selected from the same production lot. From each gown, 24 swatches were cut from the continuous chest region with a die cutting machine (Model: Mark 3, AccuCut, Omaha, NE). Individual specimens were randomly selected among the 624 swatches (26 gowns x 24 swatches per gown). Specimens were conditioned at 23 ± 2°C and 50 ± 5% relative humidity at least 24 hours prior to testing. Two polyethylene closed-cell foam gaskets were placed between each side of the fabric to provide a liquid-proof seal (SKU 8722K87, McMaster Carr, Omaha, NE). A total of 155 specimens were evaluated for liquid or viral penetration (Table 2).

**Table 2:** Summary of liquid and viral penetration tests.

	Liquid Penetration Tests	Viral Penetration Tests	
**Specimens**	n = 78	n = 57	n = 20
**Simultaneous tests**	8 specimens	1 specimen and 1 negative control	1 specimen and 1 negative control
**Challenge solution**	Nutrient broth	Nutrient broth with Phi-X174	Nutrient broth with MS2
**Filling**	Polymer tube network	Syringe	Syringe
**Environment**	23 ± 2°C and 50 ± 5% RH	23 ± 2°C and 50 ± 5% RH	23 ± 2°C and 50 ± 5% RH
**Pressure regime**	5 min. rest followed by 1.0 kPa/min	5 min. rest followed by 1.0 kPa/min	5 min. rest followed by 1.0 kPa/min
**Test end**	After 5 penetrations	At pre-determined times	At pre-determined times

### Liquid penetration tests with nutrient broth (n = 78)

Seventy-eight liquid penetration tests were conducted using a nutrient broth challenge solution (Table 3). The nutrient broth was selected to match the liquid used in the viral penetration test. By using the same challenge solution, liquid and viral tests were comparable with respect to penetrability.

**Table 3:** Nutrient broth challenge solution used for liquid and viral penetration tests.

	Identifier	Amount	Supplier
**Water**	Type 1 ultrapure	1 liter Q.S.	N/A
**Nutrient broth, powder**	Code: S25759B	8.0 g	Fisher Scientific Education, PA
**Potassium chloride**	CAS# 7447-40-7	5.0 g	ACROS, NJ
**Calcium chloride**	CAS# 100043-52-4	0.2 g	SigmaAldrich, MO
**Tween 80**	CAS# 9005-65-6	0.01%	SigmaAldrich, MO
**Fluorescein**	CAS# 518-47-8	0.752 g	ACROS, NJ

The composition of the nutrient broth was specified by ASTM F1671 [10], however, fluorescein was added to enhance visual detection of the liquid penetration. A surface tension of 48.7 ±0.5 dyn/cm was validated by a drop tensiometer (Attension One—Theta, Biolin Scientific Inc., MD). Although ASTM F1671 states that the surface tension shall be 42 ±2 dyn/cm measured by the duNouy ring, the ASTM standard test liquids were not correctly characterized [17].

A series of eight penetration cells loaded with eight fabric specimens each were filled with nutrient broth simultaneously from the bottom through a network of flexible polymer tubes. When the liquid reached the middle of the sight glass, the test cell valves were closed. Pressure was not applied for 5 minutes, then followed by a continuous and linear pressure increase of 1.0 kPa/min. Eight cameras positioned 30 cm from each penetration cell recorded the eight specimens simultaneously. Testing concluded after five penetration events were seen on each specimen. After testing, each video recording was analyzed to determine the time and location of those penetration events. The time and location of all five penetration events were recorded for future reference, however, only the first penetration event was evaluated. By using only the first penetration event, the liquid penetration data maintained a one-to-one symmetry with the viral data that cannot differentiate multiple penetration events by virtue of detection methodology (i.e. viral plating).

### Pressure head

Correction for pressure head was necessary to find the true hydrostatic pressure on a vertically oriented penetration test cell. The additional force caused by pressure head was not described in the ASTM or ISO test methods. Pressure due to liquid depth (i.e., pressure head or ψ) = p/ρg (Eq 1). By solving for fluid pressure (p), each centimeter of depth corresponded to 98 Pa of pressure induced by the gravitational force of 9.8 m/s^2^ and the density of nutrient broth, 1.0 g/mL.

ψ=p/ρg (1)

ψ is pressure head (m)

p is fluid pressure (Pa)

ρ is the density of the fluid (kg/m^3^)

g is acceleration due to gravity (9.8 m/s^2^)

### Viral penetration tests with time trials (n = 77)

For each viral test, a “sample” and a “control” were run simultaneously. The “sample” was loaded with a fabric specimen and filled with the nutrient broth challenge solution (Table 3) with the addition of Phi-174 or MS2 bacteriophage detailed in the following section. The negative “control” was filled with the same viral challenge solution, but loaded with a vinyl sheet instead of a fabric specimen. By using only two penetration cells, the viral extraction procedure began without delay and was completed within two minutes. Although Phi-X174 nucleic acid is single-stranded DNA while MS2 nucleic acid is single-stranded RNA, both bacteriophages share similar morphology: icosahedral capsid, non-enveloped, and ~27 nanometers in diameter. Contrary to the ASTM F1671 test method, the penetration cell assembly was filled from the bottom to reduce risk of viral contamination. The challenge solution was delivered into the penetration cell with a 60-mL sterile Luer-Lok syringe. After closing the valve at the bottom of the test cell, no pressure was applied for 5 minutes, followed by a continuous linear pressure increase of 1.0 kPa/min.

Viral tests (ASTM 1671 and ISO 16604) have three possible outcomes: 1) no visible penetration and no virus detected; 2) no visible penetration and virus detected; or 3) visible penetration with virus (As with ASTM F1671, visual penetration was assumed to contain virus). A 4^th^ outcome of visible liquid penetration without virus was not considered because protective clothing is not designed to filter virus.

When testing begins, we expect no penetration and no virus (outcome 1). Eventually, we expect specimens to show visible and viral penetration (outcome 3). If a test is purposely stopped prior to visible liquid penetration, we expect to see a transition from outcome 1 to outcome 3 and some specimens with no liquid penetration but containing virus (outcome 2).

Unlike liquid penetration in which events are seen, viral penetration is not visible. Because viral penetration was not visually observed, it is not possible to know the location. Therefore, there is no way to correct for gravitational head. Viral penetration is detected by extracting virus from the surface of the specimen after the pressure sequence is concluded. Time trials, i.e. stopping the test at predetermined times and evaluating the transition from outcome 1 to outcome 3, were used to determine the time of viral penetration.

Without knowing when to expect viral penetration, tests were stopped at 5, 6, 7, 8, 9, 10, 11, and 12 minutes. For the tested specimens, no virus was detected from 5 to 9 minutes (outcome 1). The specimen that was stopped at 10 minutes appeared clean but contained virus (outcome 2). The specimens that were stopped at 11 and 12 minutes had visible liquid penetration (outcome 3). The first estimate was that viral penetration occurred between 9 and 11 minutes. We then conducted 29 additional tests (Exploratory Phase) in which we stopped every 15 seconds from 7 ¼ to 12 minutes and repeated times around 10 minutes when viral penetration would likely occur. Because the stopping times in the Exploratory Phase were not randomly selected, a Validation Phase was comprised of 20 equispaced times from 8.5 to 11.35 minutes for Phi-X174 and MS2 bacteriophages (Table 4).

**Table 4:** Predetermined viral test times.

Phase	Virus	Sample Size (n = 77)	End Time
**Exploratory**	Phi-X174	n = 8	5, 6, 7, 8, 9, 10, 11, and 12 minutes
**Exploratory**	Phi-X174	n = 29	7¼ to 12 minutes, every 15 seconds with repeats
**Validation of Phi-X174**	Phi-X174	n = 20	Equispaced containing 99.7% of transitional data
**Validation of MS2**	MS2	n = 20	Equispaced containing 99.7% of transitional data

The randomly selected stop times (Validation Phases) were compared to the biased stop times (Exploratory Phase) through correlation and simple linear regression. The bivariate correlation between the cumulative distributions of viral failures between the Exploratory Phase and the Phi-X174 Phases was 0.98 leading to an adjusted R^2^ of 0.966. The bivariate correlation between the cumulative distributions of viral failures between the Exploratory Phase and the MS2 Phase was 0.97 leading to an adjusted R^2^ of 0.962. The bivariate correlation between the cumulative distributions of viral failures between the Phi-X174 and the MS2 Phase was 0.98 leading to an adjusted R^2^ of 0.966. The correlation and adjusted R^2^ suggested that the three phases of data resulted in near singular distributions of cumulative observed failures. Therefore, all three phases were not different from each other and all three phases comprised a unitary dataset of viral penetration (n = 77).

### Viral penetration test detail

The challenge suspension was prepared according to ASTM F1671. Bacteriophage Phi-X174 (ATCC 13706-B1), *Escherichia coli* (ATCC 13706), bacteriophage MS2 (ATCC 15597-B1), and *E*. *coli* (ATCC 15597) were obtained from American Type Culture Collection (ATCC, VA, USA). *E*. *coli* (ATCC 13706) and *E*.*coli* (ATCC 15597) were grown in nutrient broth according to ASTM F1671 (Table 3). Both *E*. *coli*s were incubated at 35–37°C overnight on an orbital incubator shaker at 225 revolutions/min (SteadyShake 757, Concord, CA). The next day, a 1:100 dilution of each overnight *E*.*coli* culture was prepared in 100 mL of 271 B growth medium (ATCC Medium: 271 Escherichia medium). The use of the 271 B is a departure from the ASTM F1671 standard, which uses nutrient broth only. However, 271 B provided a greater yield and contribution to the challenge solution was negligible (<0.7% volume). Each *E*.*coli* culture was grown to a density of 2 to 4 × 10^8^ CFU/mL. This cell density corresponded to a 0.3% to 0.5% absorbance reading at 640 nanometers (OrionAquaMate 8000 UV-VIS, ThermoScientific, Waltham, MA).

The *E*. *coli* (ATCC 13706) culture was inoculated with 1.0 mL of Phi-X174 bacteriophage stock (ATCC 13706-B1) having a titer of 3.6 x 10^10^ PFU/mL, a ratio of 1.8 bacteriophage to bacterial cells. The dilution was incubated at 35–37°C with shaking (225 revolutions/min) overnight then centrifuged for 20 minutes at 10,000 *g* (g-force) to remove large cell debris. The bacteriophage-containing supernatant was filtered through a 0.22-μm Millipore ExpressPlus Stericup filter. Serial dilution and plating confirmed that the final titer of the bacteriophage Phi-X174 stock was 4.2 × 10^10^ PFU/mL.

The lyophilized MS2 bacteriophage (ATCC 15597-B1) was rehydrated with 1.0 mL of 271B broth and used to inoculate the *E*.*coli* (ATCC 15597) culture. The dilution was incubated at 35–37°C with shaking (225 rpm) overnight then centrifuged for 20 minutes at 10,000 *g* to remove large cell debris. The bacteriophage-containing supernatant was filtered through a 0.22-μm Millipore ExpressPlus Stericup filter. Serial dilution and plating was used to calculate the final titer of the bacteriophage MS2 stock (9.3 × 10^10^ PFU/mL).

Compatibility testing for both Phi-X174 and MS2 was performed as described in ASTM F1671. The ratio of control assay titer over the test material assay titer (PFU/mL) was 1.4 for Phi-X174 and 1.2 for MS2. The Phi-X174 challenge suspension (150 mL) was prepared by adding 1.0 mL bacteriophage stock solution (4.2 ×10^10^ PFU/mL) in 149 mL preprepared nutrient broth and used in the penetration tests immediately. The MS2 challenge suspension (300 mL) was prepared by adding 0.9 mL bacteriophage stock solution (9.3 ×10^10^ PFU/mL) in 299.1 mL pre-prepared nutrient broth and used in the penetration tests immediately. The bacteriophage nutrient broth used in this study had a surface tension 0.048 N/m (at 20 minutes).

Criteria for pass/fail was consistent with ASTM F1671, section 15.2. If visual penetration was observed during the test, the test was terminated and without performing the assay procedure, the specimen failed. A specimen also failed if at least one PFU of virus was detected on an agar plate.

The compatibility testing of the specimen was completed before the main testing (ASTM F1671, section 15.4). Compatibility tests were conducted to show that the test apparatus contained no inhibiting substance to the bacteriophage Phi-X174 (n = 3) or bacteriophage MS2 (n = 3). Prior to each viral test, settle plates were prepared in accordance with ASTM F1671 section 13.3 and placed inside the test chamber within the vicinity of the mounted test cells. Pre- and post-challenge suspensions for each swatch run were assayed to verify no loss of Phi-X174 or MS2 bacteriophage challenge titer during the test. Duplicate plates were prepared for each assay, replicate, and negative control.

### Statistical analysis

All statistical analyses were performed with R statistical programming software [18]. To assess the temporal characteristics of liquid penetration we first checked to see if the distribution of failures formed a “bell curve.” If failures were not “normally” distributed (Shapiro Wilk statistic), the distribution of liquid penetration failures were separated into two distinct, normally distributed curves using an iterative “best fit” algorithm (mixtools package [19]).

To assess the spatial characteristics of liquid penetration the x and y coordinates of each penetration event was bounded on a two-dimensional plane enclosed by a 57.3 mm diameter circle created by the radius of the penetration cell. The data structure containing each x and y coordinate and circular bounding was defined within a ppp object (spatstat package [20]). Each penetration event was plotted and the density.ppp function was used to provide a plot of kernel smoothed intensity (i.e. penetration density). Events were further evaluated using a point pattern analysis (Kest function) to solve Ripley’s K function with a nominal 100 simulations.

To assess the time difference between liquid and viral penetration we selected a widely used logistic model for regression analysis. Because viral penetration data is non-parametric (i.e. Pass/Fail), parametric statistic regression was not an option for the viral penetration data.

## Results

### Temporal characteristics of liquid penetration events (n = 78)

**Fig 3:**
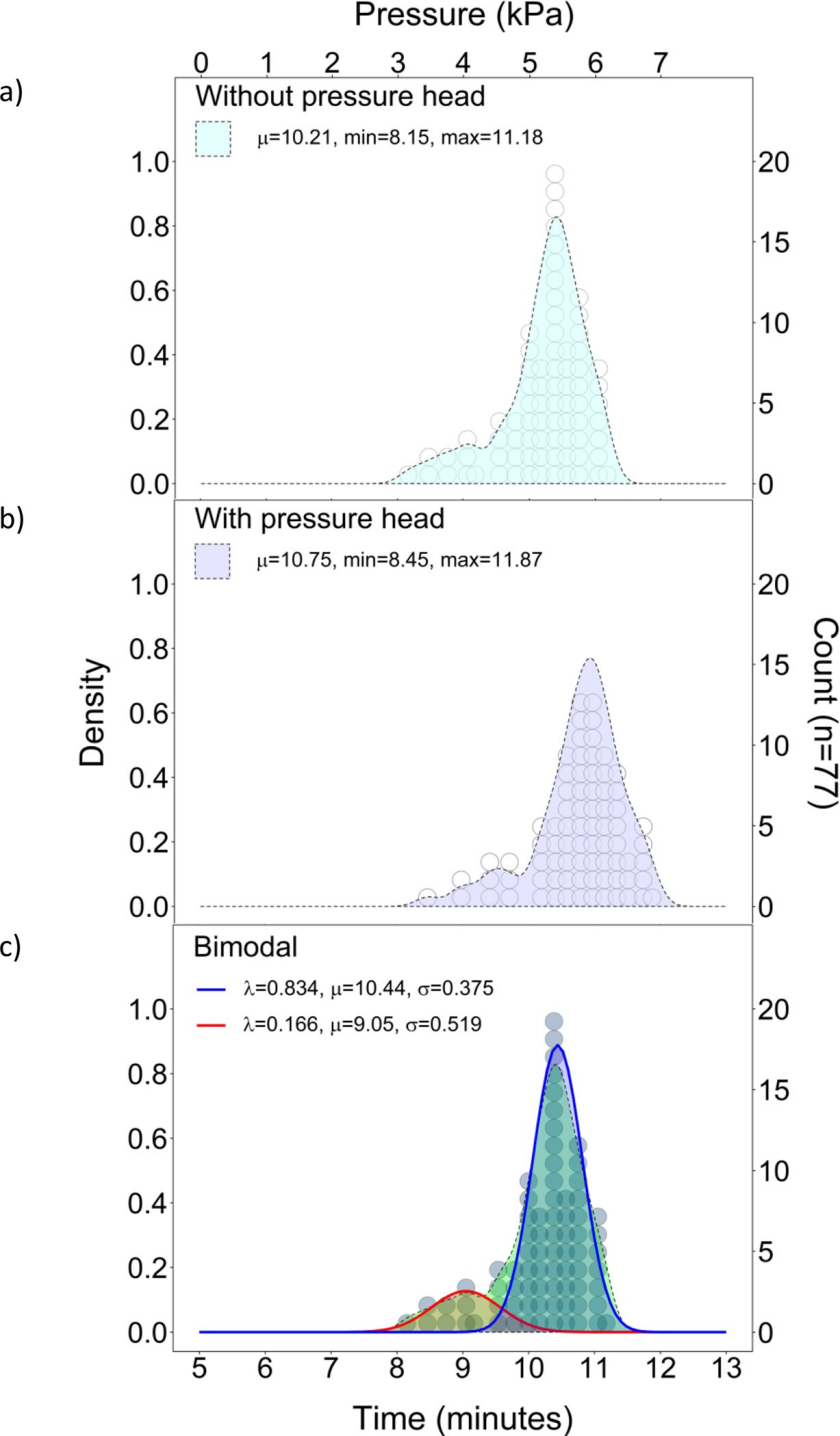
Distribution of failure times: a) without pressure head, b) with pressure head, and c) bimodal.

The liquid penetration time ranged from 8.15 to 11.18 minutes and the mean penetration time was 10.21 minutes (Fig 3A) With pressure head correction, the liquid penetration time ranged from 8.45 to 11.87 minutes and the mean penetration time was 10.75 minutes (Fig 3B). Pressure head, therefore, accounted for an increase of 248 and 773 Pa; a mean pressure increase of 545 Pa. Considering that pressure was applied at a rate of 1.0 kPa/min, pressure head was responsible for an additional 0.55 minutes of mean penetration time.

The times of liquid penetration without pressure head were not normally distributed; α<0.05%, W = 0.905, p-value = 0.001. The time of liquid penetration fit a bimodal distribution with mean penetration times of 10.44 minutes (83.4% failures) and 9.05 minutes (16.6% failures) (Fig 3C).

### Spatial characteristics of liquid penetration events (n = 78)

**Fig 4:**
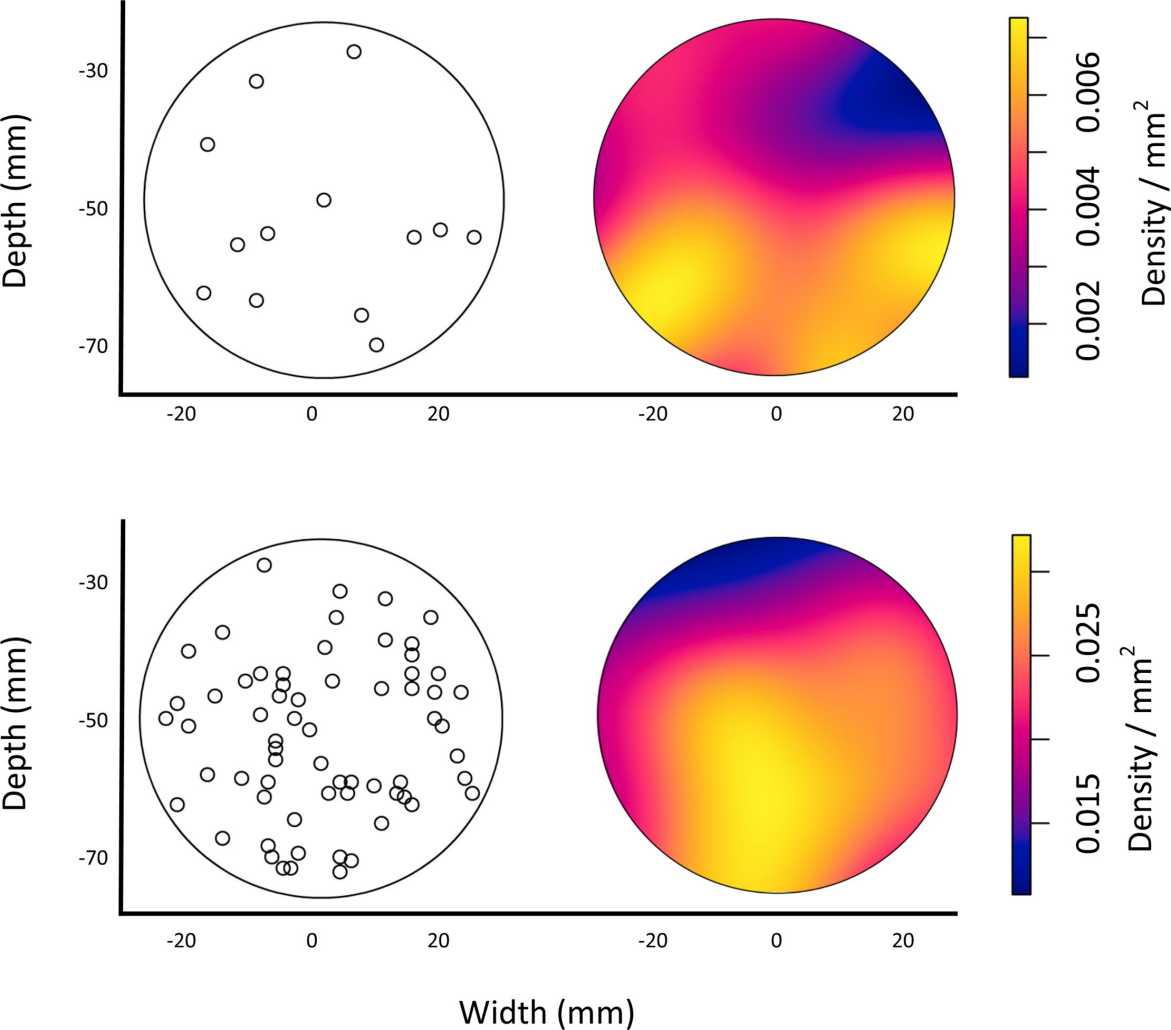
Spatial distribution of penetration events within the test apparatus for premature (n = 13) and primary (n = 65) failure modes.

To evaluate spatial distribution of the penetration events, we selected Ripley’s K function because the K(r) statistic is able to evaluate clustering over both small and large scales that would not be possible with nearest neighbor statistics. Liquid penetration events were evaluated as a full set (n = 78) and also subdivided into fail modes; premature (16.6%, n = 13) and primary (83.4%, n = 65). Ripley’s K function found that all three datasets were normally distributed when evaluated for all distances (r) ranging from 0 to 14 mm. However, density plots suggested a stratified distribution with greater density of penetration events on the bottom (Fig 4).

### Viral penetration tests with time trials (n = 77)

**Fig 5:**
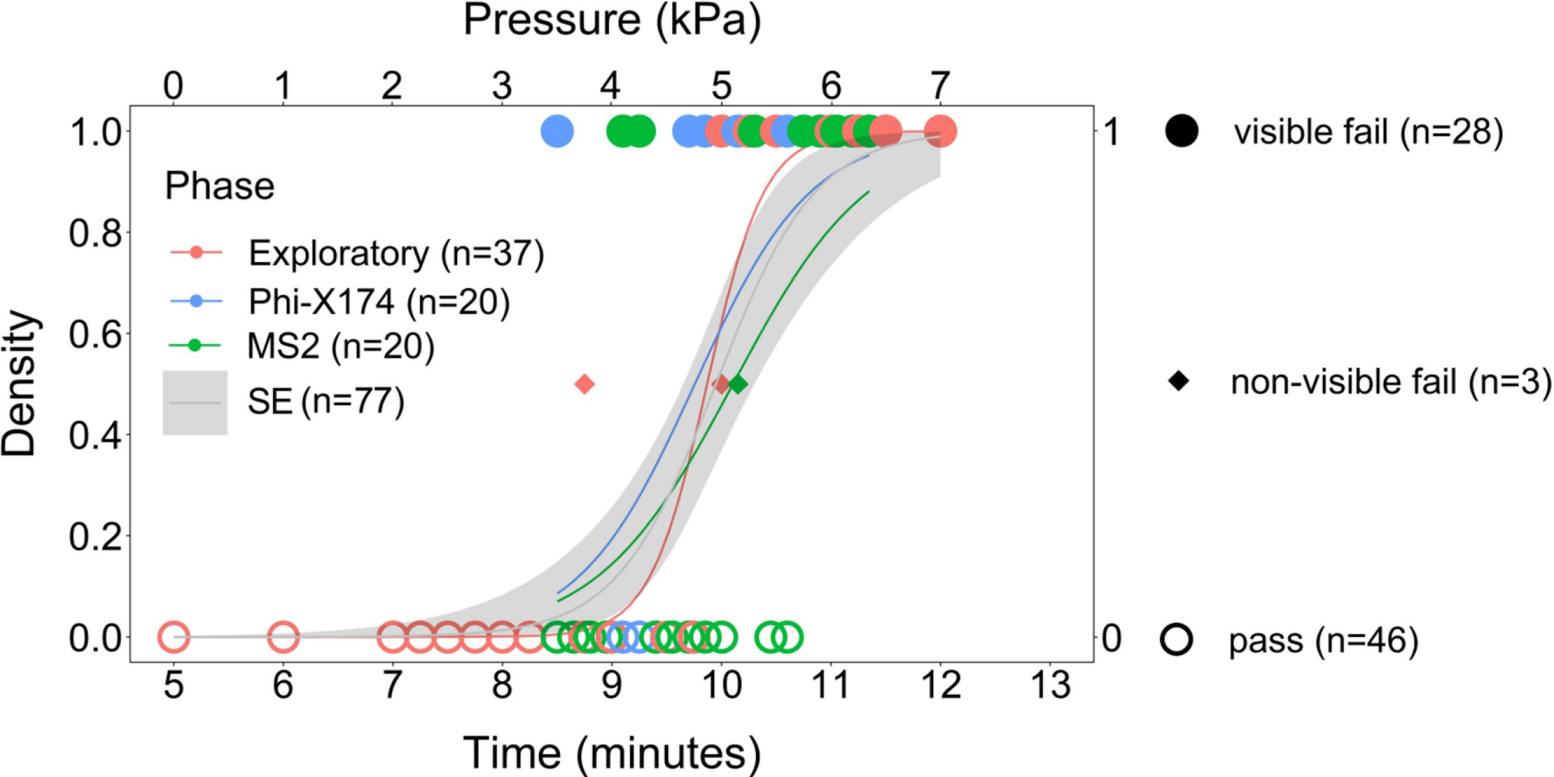
Viral penetration logistic regression of Phi-X174.

Logistic regressions of the Exploratory, Phi-X174, and MS2 Phases are shown in Fig 5. with a small number of samples, the standard error (SE) of logistic regression is large, however, the SE for the combined dataset (n = 77) are generally within the bounds of all phases. The early failures before 9.5 minutes is indicative of the early failure mode shown in Fig 3 and should to encompass 16.6% of the failures.

The three instances of non-visible virus penetration, in which virus was detected on “clean looking” specimens did not provide the minimum number of samples needed for logistic regression analysis. However, based upon the infrequency of observing the event (3 non-visible fails of 31 total fails), an estimate of the time of viral penetration was 0.29 minutes by calculating 9.7% of the duration of the 3.03 minute long penetration event.

Mean penetration times of the Exploratory, Phi-X174, and MS2 Phases were 9.87, 9.76 to 10.11 minutes, respectively. Because regression analysis showed that, the phases were unitary, these differences were attributed to variability and we, therefore, considered the mean penetration of all viral testing (9.95 minutes). Compared to the mean time of liquid penetration, 10.21 minutes (Fig 3), viral penetration was 0.26 minutes earlier than liquid penetration. However, the viral penetration failures contain mostly visible failures (n = 28) with only a few non-visible failures (n = 3). Furthermore, this value is within the SE of measurement. Therefore, we consider the previously calculated value (0.29 minutes), based on the frequency of occurrence of outcome non-visible failures (n = 3), to be a better estimate of viral penetration.

## Discussion

Temporal analysis showed a non-normal distribution of liquid penetration times between 8.15 to 11.18 minutes (n = 78). An ideal material containing equal-sized pores would have a normal distribution, while irregularities, imperfections, or calendaring the fabric may result in additional failure modes. The test apparatus itself may also cause pinching or stresses on the fabric that could result in early failures. The fabric tested had 83.4% (λ = 0.834) uniform structure with 16.6% (λ = 0.166) early failures. The magnitude of each distribution mode (83.4% and 16.6%) indicates that this fabric is mostly uniform but contains one-in-five premature failures.

Spatial analysis was used to determine if the premature failures were inherent within the fabric or were caused by stress during the test. If failures were clustered around fabric features (e.g. seams, zippers, or folds) that may indicate that the feature caused the early failures. If failures were clustered around the perimeter of the apparatus that may indicate that the apparatus or pressure had weakened the fabric. The absence of clusters indicate that the cause of early failures are likely inherent within the fabric structure.

Spatial patterns were uniformly distributed with a slight stratification in which more penetration events occurred on the bottom of the apparatus. Because gravity added pressure that increased with depth, we expected to see fewer failures at the top (Fig 4).

The time of mean viral penetration tests in all phases only slightly preceded liquid penetration. For the fabric tested, liquid penetration may therefore be a latent indicator of viral penetration. Using a very different approach, these results are in agreement with Shimasaki et. al.

Pressure control within 1% was necessary to provide accurate penetration times. The ASTM test apparatus has a manual pressure control with a tolerance of 2,758 Pa (±0.2 psi). With 1,000 Pa corresponding to one minute, the original ASTM apparatus would not be able to determine difference between liquid and viral penetration in the fabric tested.

### Limitations of the study

Continued testing on this fabric would improve statistical precision. More instances of the infrequent event (n = 3) in which the fabric appears ‘clean’ but virus is detected, may perhaps be necessary before using a liquid penetration test in lieu of a viral test for this fabric. However, the techniques presented provide an efficient approach in which we may add additional viruses or evaluate factors (e.g. other fabrics, different test liquids, and various environmental conditions).

## Conclusion

This paper presented a quantitative approach to evaluate a fabrics’ resistance to liquid and viral penetration. To our knowledge, it is the first paper to compare the time of liquid penetration to viral penetration. Testing estimated that the difference between liquid and viral penetration was 0.29 minutes for this fabric. Further evidence of the *‘viral compatibility’* between the liquid and viral test for this fabric may allow the manufacturer to substitute an inexpensive quick screening technique for a costly viral test.

The viral tests suggested that two similar viruses (Phi-X174 and MS2) did not have different viral penetration times. The technique presented here would provide an efficient way to evaluate additional viruses or additional fabrics.

All liquid and viral penetration tests were conducted on the custom modified apparatus based on ASTM F903 standard. Based upon the pressure head in vertical orientation of the penetration cell, we recommend a horizontal orientation of penetration cells to improve test accuracy. Based upon the tolerance of a manual pressure control, we recommend precision of an electronic pressure control. The temporal and spatial analysis techniques and apparatus developed in this study revealed multiple failure modes in the fabric tested. Information like this may be helpful for fabric manufacturers to improve quality control and to design garments that are more protective.

## Supporting information

S1 Datasets Test results for liquid (n = 78) and viral (n = 77) penetration events. (XLSX)
